# Cost-utility of cognitive behavioral therapy versus U.S. Food and Drug Administration recommended drugs and usual care in the treatment of patients with fibromyalgia: an economic evaluation alongside a 6-month randomized controlled trial

**DOI:** 10.1186/s13075-014-0451-y

**Published:** 2014-10-01

**Authors:** Juan V Luciano, Francesco D’Amico, Marta Cerdà-Lafont, María T Peñarrubia-María, Martin Knapp, Antonio I Cuesta-Vargas, Antoni Serrano-Blanco, Javier García-Campayo

**Affiliations:** Research and Development Unit, Parc Sanitari Sant Joan de Déu, C/Dr. Antoni Pujadas 42, Sant Boi de Llobregat, Barcelona, 08830 Spain; Primary Care Prevention and Health Promotion Research Network (RedIAPP, ISCIII), Madrid, Spain; Open University of Catalonia, Barcelona, Spain; Personal Social Services Research Unit (PSSRU), London School of Economics and Political Science (LSE), London, UK; Primary Health Centre Bartomeu Fabrés Anglada, DAP Baix Llobregat Litoral, Unitat Docent Costa de Ponent, Institut Català de la Salut, Gavà, Spain; Department of Psychiatry and Physiotherapy, Faculty of Medicine, Malaga University, Malaga, Spain; Department of Psychiatry, Miguel Servet Hospital, Aragon Institute of Health Sciences (I + CS), Zaragoza, Spain

## Abstract

**Introduction:**

Cognitive behavioral therapy (CBT) and U.S. Food and Drug Administration (FDA)-recommended pharmacologic treatments (RPTs; pregabalin, duloxetine, and milnacipran) are effective treatment options for fibromyalgia (FM) syndrome and are currently recommended by clinical guidelines. We compared the cost-utility from the healthcare and societal perspectives of CBT versus RPT (combination of pregabalin + duloxetine) and usual care (TAU) groups in the treatment of FM.

**Methods:**

The economic evaluation was conducted alongside a 6-month, multicenter, randomized, blinded, parallel group, controlled trial. In total, 168 FM patients from 41 general practices in Zaragoza (Spain) were randomized to CBT (*n* = 57), RPT (*n* = 56), or TAU (*n* = 55). The main outcome measures were Quality-Adjusted Life Years (QALYs, assessed by using the EuroQoL-5D questionnaire) and improvements in health-related quality of life (HRQoL, assessed by using EuroQoL-5D visual analogue scale, EQ-VAS). The costs of healthcare use were estimated from patient self-reports (Client Service Receipt Inventory). Cost-utility was assessed by using the net-benefit approach and cost-effectiveness acceptability curves (CEACs).

**Results:**

On average, the total costs per patient in the CBT group (1,847€) were significantly lower than those in patients receiving RPT (3,664€) or TAU (3,124€). Patients receiving CBT reported a higher quality of life (QALYs and EQ-VAS scores); the differences between groups were significant only for EQ-VAS. From a complete case-analysis approach (base case), the point estimates of the cost-effectiveness ratios resulted in dominance for the CBT group in all of the comparisons performed, by using both QALYs and EQ-VAS as outcomes. These findings were confirmed by bootstrap analyses, net-benefit curves, and CEACs. Two additional sensitivity analyses (intention-to-treat analysis and per-protocol analysis) indicated that the results were robust. The comparison of RPT with TAU yielded no clear preference for either treatment when using QALYs, although RPT was determined to be more cost-effective than TAU when evaluating EQ-VAS.

**Conclusions:**

Because of lower costs, CBT is the most cost-effective treatment for adult FM patients. Implementation in routine medical care would require policymakers to develop more-widespread public access to trained and experienced therapists in group-based forms of CBT.

**Trial registration:**

Current Controlled Trials ISRCTN10804772. Registered 29 September 2008.

**Electronic supplementary material:**

The online version of this article (doi:10.1186/s13075-014-0451-y) contains supplementary material, which is available to authorized users.

## Introduction

Fibromyalgia (FM) is a complex clinical entity that is currently considered to be part of the spectrum of central sensitivity syndromes [1]. FM was defined in 1990 as the presence of chronic widespread pain lasting for more than 3 months and patient reports of tenderness in at least 11 of 18 defined tender points when digitally palpated with approximately 4 kg per unit area of force [2]. In May 2010, the American College of Rheumatology (ACR) published preliminary diagnostic criteria that eliminate the tender-point examination [3].

According to a recent review [4], the prevalence estimates of FM in the general population have ranged from approximately 1% to 11%, with women being considerably more vulnerable to FM than men. The prevalence of the syndrome increases with age until approximately the sixth decade and decreases thereafter.

Several studies have analyzed the health-care and societal burdens associated with FM in industrialized countries [5,6]. Leadley and colleagues [5] reviewed 10 cost and 29 prevalence studies and noted that among chronic-pain conditions, FM syndrome had the highest unemployment rate (6%), highest claim rate for incapacity benefits (from 11.9% to 29.9%), and greatest number of days absent from work (from 21 to 73).

Currently, no curative treatments are available for patients with FM. Both pharmacologic and nonpharmacologic approaches are used by clinicians to alleviate the constellation of FM symptoms and to improve patients’ functioning. To date, only the following medications have been approved by the US Food and Drug Administration (FDA) for FM pain: pregabalin (second-generation anticonvulsant), approved in 2007; duloxetine (SNRI), approved in 2008; and milnacipran (SNRI), approved in 2009. In contrast, the European Medicines Agency (EMA) refused to approve these medications, based on a benefit-risk assessment [7-9].

A recent meta-analysis [9] of eight RCTs that focused on the effectiveness of anticonvulsants yielded the following results: a slight reduction of pain and sleep problems with pregabalin compared with placebo after 13 weeks of treatment, on average. The results suggested that the effects of pregabalin in reducing fatigue, depression, and anxiety and in improving HRQoL were limited. Additionally, the reported side effects from the use of pregabalin included dizziness.

Häuser and colleagues [10] revised and performed a meta-analysis of 10 studies to ascertain the benefits and harms of FDA-recommended SNRIs compared with a placebo when treating FM symptoms in adults. The results suggested that duloxetine and milnacipran only had a small incremental effect over the placebo in reducing pain. The effect on fatigue was not substantial, and both were inferior to the placebo for reducing sleep problems. Finally, Nüesch *et al.* [11] performed a network meta-analysis of randomized trials in patients with FM that evaluated the effects of pharmacologic and nonpharmacologic interventions recommended by FM guidelines. The authors found statistically significant advantages of SNRIs and pregabalin over placebo on pain and quality of life, although these benefits were not clinically relevant. They concluded that benefits of pharmacologic treatments in FM were of questionable clinical relevance, and evidence for the benefits of nonpharmacologic interventions is limited. This conclusion about the modest effectiveness of pharmacologic and nonpharmacologic approaches might be generalized to all chronic pain conditions [12,13].

Concerning nonpharmacologic interventions, Bernardy *et al*. [14] performed a meta-analysis of 23 studies that indicated that treatment with CBTs provided a small incremental benefit over control interventions in reducing pain, negative mood, and disability at end of treatment and at long-term follow-up (median, 6 months) in patients with FM. Overall, the RCTs had poor quality, but the sensitivity analyses demonstrated that the results were robust against the risks of bias.

Although the aforementioned meta-analyses provided data about the effectiveness of specific pharmacologic and nonpharmacologic treatments for FM, evidence is lacking regarding their cost-effectiveness [15]. In the current worldwide context of economic crisis, decision-makers are faced with health-care budget constraints and must prioritize their expenditures on the pharmacologic or nonpharmacologic option that is the most cost effective for the treatment of a particular patient group. A cost-effectiveness analysis can help to resolve the dilemma of which available treatment generates the maximal health benefits for the health care system and for society as a whole.

Two studies have examined the cost-effectiveness of pregabalin in the treatment of FM, one from a UK perspective [16] and one from a US perspective [17]. Choy *et al*. [16] analyzed the cost-effectiveness of 300 mg and 450 mg of pregabalin in the treatment of FM compared with placebo, duloxetine, gabapentin, tramadol, and amitriptyline. Pregabalin was found to be cost-effective at a threshold of £30,000 per QALY gained compared with the other treatment options or placebo in approximately 60% of patients participating in FM clinical trials. Pregabalin, 450 mg, was consistently more cost-effective than pregabalin, 300 mg. More recently, Lloyd *et al.* [17] replicated the analysis, but from the US perspective. Pregabalin was less costly than placebo for both direct and indirect costs. In this model, pregabalin, 450 mg, was consistently more cost-effective than pregabalin, 300 mg. Beard *et al.* [18] carried out an economic evaluation of the cost-effectiveness of 60 mg/day duloxetine. The analyses revealed that it might be more cost-effective to consider duloxetine as a second-line treatment option, after failure on tricyclic antidepressants.

Among nonpharmacologic treatments [19-22], only psychoeducation [22] has shown evidence of cost-effectiveness. Luciano *et al*. [22] found that usual care complemented by a 9-week psychoeducation program was effective and cost-effective for FM within a general-practice setting and at a 12-month follow-up. Only Alda *et al*. [23] compared the effectiveness of CBT versus FDA-recommended pharmacologic treatment (RPT, pregabalin + duloxetine in case of comorbid major depression) and treatment-as-usual (TAU). The 6-month follow-up evaluation revealed that CBT was more effective than RPT, taking a wide range of clinical variables into account.^a^ In a recent review [24], it was highlighted that a web-based CBT program (“Living Well with Fibromyalgia”) demonstrated effect sizes in pain and physical functioning that were comparable or superior to the FDA-approved medications. The numbers needed to treat were 7.2, 19.0, and 8.6 for duloxetine, milnacipran, and pregabalin, respectively; but only 5 for the web-based CBT program.

In the present work, we extend our previously reported findings of CBT in FM [23] by comparing, for the first time, the 6-month healthcare and societal costs associated with CBT, FDA-RPT, and TAU, as well as the 6-month cost-effectiveness of CBT, FDA-RPT, and TAU in terms of gains in QALYs and increases in HRQoL.

## Methods

A detailed description of the RCT protocol and the effectiveness results can be reviewed elsewhere [23,25]^b^. This study was performed in accordance with the ethical standards established in the 1964 Declaration of Helsinki and its subsequent updates and established in the Declaration of Madrid of the World Psychiatric Association. The study protocol was approved through the ethical review board of the Aragon Health Sciences Institute (IACS), Aragon, Spain (February 2007; ETES number PI07/90959). The IACS is the center responsible for research and knowledge management in Biomedicine and Health Sciences in the public Aragon Health System. All participants provided written informed consent before the commencement of any study activities or procedures.

This study followed the Initiative on Methods, Measurement, and Pain Assessment in Clinical Trials (IMMPACT) recommendations for chronic pain clinical trials [26]; the Consolidated Standards of Reporting Trials (CONSORT) recommendations for randomized, controlled trials [27]; and the Consolidated Health Economic Evaluation Reporting Standards (CHEERS) statement [28] [see Additional file 1].

### Design

The study was a 6-month, multicenter, randomized, parallel-group, controlled trial in which patients with FM were recruited from any of the 41 primary healthcare centers in the city of Zaragoza (Aragon, Spain) and randomly assigned to one of three study groups (ratio, 1:1:1): CBT (*n* = 57), RPT (*n* = 56), and TAU at the primary health care level (*n* = 55). The two evaluators (clinical psychologists) were blinded to patients’ treatment-group assignments.

Patients considered for inclusion were adults from 18 to 65 years of age who were able to understand and read Spanish, fulfilled the 1990 ACR classification criteria [2] for FM, had undergone no psychological treatment during the preceding 2 years, were receiving no pharmacologic treatment at that time or were willing to discontinue it for 2 weeks before the start of the study, and had signed an informed-consent statement. Those excluded were patients with severe axis I psychiatric disorders (dementia, schizophrenia, paranoid disorder, and alcohol and/or drug abuse); patients with severe axis II psychiatric disorders or other medical conditions that, from the clinician’s point of view, prevented the patient from following the treatment protocol; women who were pregnant or nursing; and those who declined to participate.

Aragon is one of the 17 regions or “autonomous communities” of Spain. As a consequence of a devolution process that started in 1981, the autonomous communities have full governance of health and social care. Health care is publicly financed, with universal coverage. The Aragon Health Care System covers all of the region’s territory. (The region of Aragon has more than 1,200,000 inhabitants). Social care is also covered for people with a functional dependency due to severe disability.

### Interventions

An outline of the nine CBT group sessions is displayed in Table 1. The CBT intervention was highly structured and performed in a group-based format (maximum of eight patients per group). Throughout the CBT treatment, extensive emphasis occurred on putting into practice skills outside of sessions. In each session, patients were assigned homework tasks with the goal of fostering practice of the skills outside of the sessions. It was considered essential that FM patients practice skills outside of sessions, as part of their daily lives, so they could become more comfortable with new, unfamiliar behaviors and ways of thinking, learn what types of coping strategies work best for them, and, important, have the opportunity to identify automatic, negative thoughts and cognitive errors, and to review problems in implementing skills outside the safe environment of the CBT group.

**Table 1:** **Session outlines for the Cognitive Behavioral Therapy (CBT) group treatment protocol**

**Session**	**Group CBT**
1	Discussing the connection between stress and pain
2	Identification of automated, negative thoughts
3	Evaluation of automated, negative thoughts
4	Challenging the automatic, negative thoughts and constructing alternatives
5	Nuclear beliefs
6	Nuclear beliefs about pain
7	Changing coping strategies
8	Coping with ruminations-obsessions-worrying. Session focused on pain catastrophizing.
9	Expressive writing and assertive communication

Trained clinicians at the Torrero health center administered the intervention. Random sessions were audio-recorded to confirm that the CBT program was exclusively delivered. Although CBT is usually considered a complement (adjuvant) to usual medical treatment, not a substitute for it, we decided to test its effectiveness as a stand-alone intervention. Therefore, only minor co-medication was allowed in the CBT group (occasionally minor analgesics), but no pregabalin, gabapentin, opioids, or antidepressants were permitted.

Regarding the RPT, pregabalin (300 to 600 mg/day) and duloxetine (60 to 120 mg/day in case of comorbid major depression) was administered to FM patients. One psychiatrist monitored RPT throughout the study.

Finally, the TAU group received the standard care delivered by general practitioners (GPs) at their primary healthcare centers. To improve this treatment, GPs received the *Guide for the Treatment of Fibromyalgia in Primary Care,* which is edited and distributed by the Aragonese Health Service. In Spanish primary health care, the treatment administered to FM patients is mainly pharmacologic and is adjusted to the symptomatic profile of the individual. In addition, counseling regarding aerobic exercise adjusted to patients’ physical levels is usually provided. TAU means that GPs selected a pharmacologic treatment and the frequency of patient visits that they considered adequate. Neither the RPT patients nor the TAU patients received any psychological intervention during the 6-month trial.

**Figure 1:**
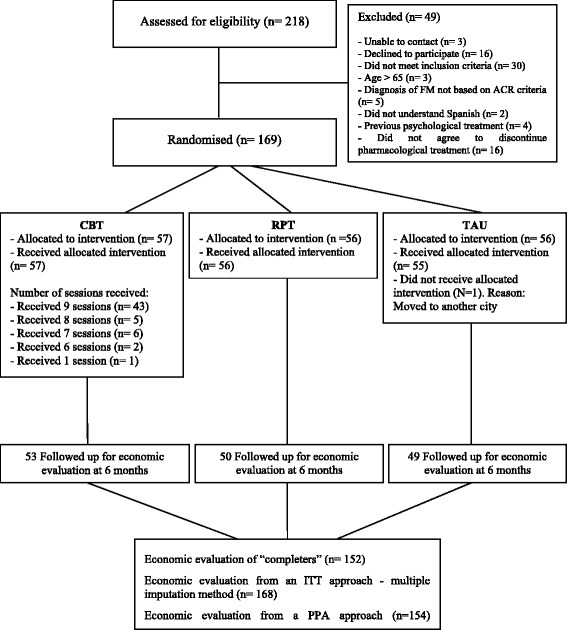
**Flow chart of the economic evaluation.**

A comprehensive summary and evaluation of the study was recently published by Bernardy and colleagues [14], who assigned 7 points to the trial by using Yates rating scale [29], a score that indicates a high treatment quality. Figure 1 illustrates the flow of participants through the economic evaluation, and Table 2 shows the baseline sociodemographic and clinical characteristics of the participants by treatment group. No statistically significant differences were noted between the three study conditions in any sociodemographic or clinical variable at baseline.

**Table 2:** **Baseline sociodemographic and clinical characteristics of the study participants by treatment group**

**Sociodemographic variables**	**CBT**	**RPT**	**TAU**	***P***
	**(n =57)**	**(n =56)**	**(n =55)**	
**Gender, females, n (%)**	54 (94.7%)	52 (92.9%)	53 (96.4%)	0.71
**Mean age, years (±SD)**	46.35 (6.71)	47.12 (6.25)	47.04 (6.53)	0.79
**Marital status, n (%)**				0.99
Married or in a relationship	40 (70.2%)	40 (71.4%)	37 (67.3%)	
Single	9 (15.8%)	9 (16.1%)	10 (18.2%)	
Separated or divorced	8 (14%)	7 (12.5%)	8 (14.5%)	
Widowed	0 (0%)	0 (0%)	0 (0%)	
**Ethnic group, n (%)**				1.00
European	57 (100%)	56 (100%)	55 (100%)	
**Living arrangement, n (%)**				0.99
Living alone	4 (7.0%)	4 (7.1%)	6 (10.9%)	
Living with spouse or partner	8 (14.0%)	9 (16.1%)	8 (14.5%)	
Living with offspring and/or spouse/partner	34 (59.6%)	30 (53.6%)	31 (56.4%)	
Living with other relatives	5 (8.8%)	7 (12.5%)	5 (9.1%)	
Other	6 (10.5%)	6 (10.7%)	5 (9.1%)	
**Educational level, n (%)**				0.81
Illiterate	0 (0%)	0 (0%)	0 (0%)	
Primary school	23 (40.4%)	23 (41.1%)	28 (50.9%)	
Secondary school	23 (40.4%)	22 (39.3%)	18 (32.7%)	
University	11 (19.3%)	11 (19.6%)	9 (16.4%)	
**Employment status, n (%)**				0.99
Unemployed	19 (29.8%)	15 (26.8%)	15 (27.3%)	
Paid employment	9 (15.8%)	11 (19.6%)	9 (16.4%)	
On sick leave from paid employment	13 (22.8%)	12 (21.4%)	14 (25.5%)	
Retired/pensioner	7 (12.3%)	6 (10.7%)	8 (14.5%)	
Permanent disability	11 (19.3%)	12 (21.4%)	9 (16.4%)	
**Income**				0.40
<MS (600€/month)	15 (26.3%)	15 (26.8%)	27 (49.1%)	
1 to 2 MS	24 (42.1%)	23 (41.1%)	20 (36.4%)	
>2 to 4 MS	18 (31.6%)	18 (32.1%)	8 (14.5%)	
>4 MS	0 (0)	0 (0)	0 (0)	
**Clinical variables**				
Mean years since FM diagnosis (±SD)	12.91 (7.15)	11.23 (3.85)	11.69 (4.02)	0.22
Preference for psychotherapy, n (%)	28 (49.1%)	26 (46.4%)	27 (49.1%)	0.95
Comorbid major depressive disorder, n (%)	27 (47.4%)	26 (46.4%)	30 (54.5%)	0.65
Sexual abuse, n (%)	4 (7.0%)	7 (12.5%)	11 (14.5%)	0.43
Currently engaged in litigation, n (%)	17 (29.8%)	12 (21.4%)	16 (29.1%)	0.54

CBT, cognitive behavioral therapy; MS, minimum salary; RPT, recommended pharmacologic treatment; TAU, treatment as usual.

### Instruments

The participants completed the following assessments as part of a paper-and-pencil battery of measures:

The *Sociodemographic questionnaire* collected information on the following variables: gender, date of birth, marital status, living arrangements, education level, and employment status (which was asked at all assessment time points).

The MINI Neuropsychiatric Interview (M.I.N.I v5.0) [30,31] is a brief, structured diagnostic interview designed to assess DSM-IV and ICD-10 psychiatric disorders. The M.I.N.I. is the most widely used psychiatric structured diagnostic interview in the world. In the present study, we specifically assessed the presence of Axis I psychiatric disorders (dementia, schizophrenia, paranoid disorder, alcohol and/or drug use disorders).

The EuroQoL-5D questionnaire (EQ-5D) [32] is a widely used health-related quality-of-life instrument with a non-disease-specific classification system composed of two parts: Part 1 is a self-reported description of health problems according to a five-dimensional classification (mobility, self-care, usual activities, pain/discomfort and anxiety/depression). Patients mark one of three levels of severity (1 = no problems, 2 = some/moderate problems, and 3 = severe/extreme problems) in each dimension. The time frame is the day of responding. Combinations of these categories define a total of 243 (3^5^) different health states. Part 2 records the current subject's health on a Visual Analogue Scale (VAS); it consists of a visual scale graded from 0 to 100, in which the respondent can self-report the current health status, with 100 being the best imaginable health level. The EQ-5D has already been demonstrated to be a valid and reliable tool to assess health outcomes of the Spanish population [33].

The *Client Service Receipt Inventory–Spanish version* (CSRI) [34] variation used in this study was designed to collect retrospective data on medication and service receipt.

*Medication use*. A profile of the patient's use of some prescribed medications (analgesics, short- and long-acting opioids, anticonvulsants, antidepressants, and so on) was requested, including the name of the drug, the prescriber, the dosage level, the total number of prescription days for the drug, the daily dosage consumed, the reasons for changing the drug, and adherence.

*Service receipt*. The main categories were: emergency service (total visits), general medical in-patient hospital admissions (total days), and outpatient health care services (total visits to GP, nurse, social worker, psychologist, and other community health care professionals). Each service was recorded as being provided by the public or by the private sector. Patients were also asked about the type and number of diagnostic tests administered. The CSRI was administered on two occasions with equal time frames: at baseline and at a 6-month follow-up; at both occasions, the previous 6 months were reviewed. Empiric evidence indicates that data obtained by the self-report has equal validity to register-collected data [35].

### Statistical analyses

#### Description of the costing procedure

Costs were estimated from the healthcare and societal perspectives during the 6 months of follow-up. *Direct health care costs* were calculated by adding the costs derived from medication consumption, medical tests, use of health-related services, and cost of the staff running the CBT intervention. The cost of medication was calculated by determining the price per milligram according to the Vademecum International (Red Book; edition 2011) and included the value-added tax. The total costs of medications were calculated by multiplying the price per milligram by the daily dosage used (in milligrams) and the number of days that the treatment was received.

The main source of the unit-cost data for medical tests and health services use was the SOIKOS database of health care costs [36]. The SOIKOS database contains information about Spanish healthcare service costs and was derived by systematic reviews of the literature; it consists of approximately 18,000 entries. The calculation of the total cost of CBT intervention per patient was based on the price per hour of a clinical psychologist, established by the Official College of Psychologists of Spain. *Indirect costs* (lost productivity): Lost productivity was calculated by using the human capital approach, which involves multiplying the minimum daily wage in Spain for 2011 by the number of days of sick leave, as reported by each patient.

Finally, *total costs* were calculated by adding the direct and indirect costs. Unit costs are expressed in Euros (€) based on 2011 prices. Table 3 shows the unit costs of healthcare resources. The time horizon was less than a year; therefore, it was not necessary to apply a discount factor to the costs.

**Table 3:** **Unit costs used in the calculations of direct healthcare costs and indirect costs (year 2011 values in €)**

**Type of use**		**Unit costs**
**Costs in the health care system (public/private)**	General practitioner	10.5/27.5
	Nurse or psychiatric nurse	10.0/25.4
	Social worker	14.9/25.4
	Psychologist	70.6/70.6
	Psychiatrist	42.9/42.9
	Other medical specialists	51.1/51.1
	Hospital emergency visits	155.5
	Hospital stay	617.5
	Diagnostic tests (range)	4.3 to 434.6
	Pharmacologic treatment	Depending on type and dose
**Productivity losses**	Absenteeism from work (minimum and medium daily wage)	21.4 to 62.7
**CBT costs**	Cost of the group intervention per session (eight patients/group; nine sessions)	240

#### Utility scores

Patients described their quality of life by using the EQ-5D questionnaire, which represents a preferable alternative when implementing economic evaluations with a societal perspective. Utility scores obtained from the questionnaire are used to rate patients’ quality of life on a scale from 0 (as bad as death) to 1 (perfect health). Negative values are possible and indicate a health state that is “worse than death”. QALYs were calculated on the basis of these scores by using the Spanish tariffs of EQ-5D [37]. Along with EQ-5D utility scores, scores recorded on the EQ VAS were also used as an outcome for the analysis.

#### Cost-utility analysis

Cost-utility was explored through the calculation of incremental cost-effectiveness ratios (ICERs), defined as the ratio between incremental costs and incremental effects measured on QALYs or EQ VAS [38]. QALYs were approximated by using the area-under-the-curve technique.

We estimated incremental marginal costs and incremental effects with the SUR (seemingly unrelated regression) model by using STATA. Cost and outcome measures were therefore included in a bivariate system that implemented a regression of costs and QALYs (or EQ VAS) on treatment allocations (that is, whether they were assigned to CBT, RPT, or TAU).

The regressions controlled also for the following variables at baseline: age, gender, marital status, education level, living arrangement, employment status, minimum wage, duration of the illness (in years) since the first diagnosis, baseline costs, and baseline outcome, depending on the equation considered.

Estimates were run by using 1,000 bootstrap replications to address a possible skewedness in the distribution of the dependent variables [39]. By using a series of hypothetical values for willingness-to-pay (λ) for one additional unit of outcome (QALY), net-benefits (NBs) were calculated. We plotted NB curves to compare the Net Monetary Benefit (NMB; y-axis) obtained by the three alternative interventions with the corresponding willingness-to-pay (WTP; x-axis).

Cost-effectiveness acceptability curves (CEACs) will also be plotted when necessary [40]. CEACs display the probability that the intervention is cost-effective, given a varying threshold for the willingness to pay for each QALY gained. The curves obtained incorporate the uncertainty that exists around the estimates of incremental costs and incremental effects associated with the intervention [41].

First, we did a complete case analysis without the 16 FM patients who were lost at 6-month follow-up. Second, the cost-utility analysis was repeated after an intention-to-treat (ITT) approach (first sensitivity analysis). The way in which missing data are handled is of crucial importance when assessing the results of economic evaluations. For the 6-month follow-up evaluation, a small number of missing values (9.5%) were imputed. We assumed data to be missing at random (MAR). Multiple imputation methods according to the chained-equations approach were used to impute missing values for the EQ-5D domains and for the costs of the nonresponders at 6 months [42-45]. The imputation model, run on 10 imputed datasets, included important sociodemographic and prognostic variables associated with the outcome variables and dropouts.

Finally, we also performed a per-protocol analysis (PPA; 2^nd^ sensitivity analysis) in which the 14 FM patients who did not attend the nine CBT sessions were excluded.

All statistical analyses were performed by using STATA version 13.0 (Stata Corp. College Station, TX, USA).

## Results

### Costs

In Table 4, we display descriptive statistics for different groups of costs, along with the adjusted and unadjusted *P* values by treatment group, as obtained with bootstrapped *t* tests (1,000 replications). None of the differences at baseline was found to be statistically significant. The average cost of primary care services for 6-month use was found to be approximately 100€ at baseline for each of the treatment groups. However, although the average cost of primary care services remained roughly at the same level at 6 months after baseline, the use related to individuals from the CBT group diminished to approximately 80€, whereas the use by members of the other two groups increased to approximately 110€. The difference at this stage was found to be significant both with the un-adjusted and adjusted *P* values.

**Table 4:** **Summary statistics of the costs (total and disaggregated in components) and outcomes by treatment group**

	**CBT**		**RPT**		**TAU**		**Significance test**	
	**Mean**	**SD**	**Mean**	**SD**	**Mean**	**SD**		
**Before baseline (6 months before baseline) -** ***N*** **=168**	***n*** **=57**		***n*** **=56**		***n*** **=55**		***P*** **value**	**adj.** ***P*** **value**
**Costs (€)**								
Primary health care services	102.7	21.9	103.1	21.5	104.3	14.5	0.87	0.99
Specialized healthcare services	1554.9	3499.8	1150.0	3113.1	1038.3	2813.4	0.67	0.55
Medical tests	49.2	113.7	48.2	103.8	48.5	101.4	1.00	0.97
Prescribed medications	475.7	876.6	563.0	1139.3	581.2	1196.7	0.83	0.80
Total direct costs	2182.4	3609.6	1864.3	3232.8	1772.3	2954.4	0.79	0.71
Total indirect costs	916.3	1415.3	741.8	1379.5	771.2	1335.5	0.77	0.81
Total costs	3098.8	3999.7	2606.1	3871.2	2543.5	3486.3	0.71	0.62
**Outcomes**								
EQ-5D Utility score	0.40	0.26	0.40	0.27	0.38	0.27	0.93	1.00
EQ VAS	45.18	16.98	46.79	15.48	43.36	14.50	0.50	0.64
**Follow-up (0 to 6 months),** ***N*** **=152**	***n*** **=53**		***n*** **=50**		***n*** **=49**		***P-*** **value**	**adj.** ***P*** **-value**
**Costs (€)**								
Primary health care services	80.9	35.4	110.8	19.6	112.6	19.5	0.00	0.00
Specialized healthcare services	940.2	2731.7	1854.4	4109.2	1663.5	3612.7	0.32	0.01
Medical tests	44.7	115.8	67.3	119.2	65.9	110.6	0.54	0.00
Prescribed medications	33.0	55.3	828.1	356.5	530.8	322.0	0.00	0.00
Intervention CBT	271.1	24.7	0.0	0.0	0.0	0.0	0.00	0.00
Total direct costs	1,369.9	2,738.5	2,860.6	4,161.3	2,372.8	3,570.2	0.07	0.00
Total indirect costs	476.8	887.6	803.0	1,307.6	750.9	1,226.3	0.21	0.00
Total costs	1,846.7	2,942.9	3,663.7	4,539.1	3,123.7	3,952.5	0.03	0.00
**Outcomes**								
EQ-5D Utility score	0.61	0.25	0.53	0.27	0.54	0.28	0.28	0.13
EQ VAS	59.62	15.78	57.30	14.11	52.86	14.25	0.07	0.00
QALY (based on EQ-5D utility score)	0.25	0.12	0.23	0.13	0.24	0.13	0.63	0.13

The cost of specialized health care services was found to be higher at baseline for the CBT group (1,550€), with the RPT (1,150€) and TAU (1,040€) groups showing lower averages. In contrast, at the 6-month follow-up, the cost of specialized health care services diminished to 940€ for the CBT group, whereas it increased considerably for the RPT (1,850€) and for the TAU groups (1,660€). The adjusted difference between groups was found to be significant.

The cost of medical tests was found to be approximately 50€ at baseline for all groups, although it increased at follow-up to 67€ and 66€ for the RPT and for the TAU groups, respectively. Both groups were found to be significantly different at follow-up compared with the CBT group (<45€).

The cost of prescribed medications at baseline was found to be lower for the CBT group (475€), whereas it was higher for the RPT (563€) and for the TAU (581€) groups. At follow-up, the use of medications by individuals in the CBT group decreased to an average of 33€, which is attributed mainly to the nature of the intervention that required individuals from this group to discontinue the use of most medications. The use of medications from the other two groups instead was increased for RPT (828€) and was slightly reduced for TAU (530€).

Overall, the direct costs were higher for the CBT group at baseline (2,200€) with respect to the RPT (1,864.3€) and TAU groups (1,772.3€). However, at follow-up, the average cost for CBT reduced to 1,370€, whereas it increased for RPT (2,860€) and TAU (2,370€). The difference at this stage was found to be significant with the adjusted *P* value.

A similar pattern was observed for indirect costs, which were initially slightly higher for the CBT group (approximately 900€) but were halved at follow-up (approximately 480€) to a level significantly lower than for the other two groups (see adjusted *P* value). Total costs were consequently lower at follow-up for the CBT group (1,850€) than for the RPT (3,660€) and TAU (3,100€) groups, reverting the situation that was found at baseline.

### Outcomes

Table 4 also shows the descriptive statistics of the EQ-5D utility and EQ VAS as well as of QALY based on the EQ-5D utility at follow-up. Utility scores for all of the groups increased from an average of 0.40 to an average of 0-50-0.60, with individuals in the CBT group showing the highest scores (small differences that were not statistically significant). A similar situation was found for the EQ VAS, for which the three groups presented similar values at baseline (approximately 45), but for which the CBT group reported better results than the other groups at follow-up (approximately 60). We can observe that all of the groups showed improvement in their EQ-5D indicators at follow-up. QALYs at follow-up, calculated for the 6-month intervention period, were found to be similar for all groups, with no significant differences.

### Cost-utility analyses

#### Societal perspective

Table 5 shows the ICERs for the comparison of the three treatment groups. As we explained earlier, the main analysis focuses on the sample of “Completers” (*N* = 152); we also performed two alternative sensitivity analyses by using the ITT sample, which includes all of the patients assessed at baseline (*N* = 168) and the PPA sample (*N* = 154), which excludes the 16 individuals who did not attend all nine CBT sessions.

**Table 5:** **Incremental cost-effectiveness ratios, societal perspective, 0 to 6 months**

	**Incremental cost**			**Incremental effect**			**ICER**
	**Mean; (95% bootstrap CI)**			**Mean; (95% bootstrap CI)**			
**CBT versus TAU**							
**Completers (** ***N*** **=152)**							
QALY (EQ-5D)	−2,061.9	−3,168.9	−954.9	0.01	−0.00	0.03	**CBT dominant**
EQ VAS	−2,073.2	−3,179.8	−966.6	6.19	3.63	8.75	**CBT dominant**
**ITT (** ***N*** **=168)**							
QALY (EQ-5D)	−2,158.8	−3,357.1	−960.5	0.02	−0.00	0.03	**CBT dominant**
EQ VAS	−2,163.9	−3,363.8	−964.0	6.33	3.62	9.07	**CBT dominant**
**PPA (** ***N*** **=154)**							
QALY (EQ-5D)	−2,368.9	−3,599.1	−1,138.6	0.02	−0.00	0.03	**CBT dominant**
EQ VAS	−2,372.4	−3,604.8	−1,140.0	6.58	3.83	9.33	**CBT dominant**
**CBT versus RPT**							
**Completers (** ***N*** **=152)**							
QALY (EQ-5D)	−2,386.9	−3,485.4	−1,288.3	0.01	−0.00	0.03	**CBT dominant**
EQ VAS	−2,393.9	−3,495.2	−1,292.6	3.52	1.13	5.90	**CBT dominant**
**ITT (** ***N*** **=168)**							
QALY (EQ-5D)	−2,304.7	−3,326.6	−1,282.7	0.01	−0.00	0.03	**CBT dominant**
EQ VAS	−2,307.8	−3,331.4	−1,284.2	3.61	1.08	6.15	**CBT dominant**
**PPA (** ***N*** **=154)**							
QALY (EQ-5D)	−2,550.3	−3,624.9	−1,475.7	0.01	−0.00	0.03	**CBT dominant**
EQ VAS	−2,552.7	−3,631.5	−1,473.9	3.93	1.35	6.51	**CBT dominant**
**RPT versus TAU**							
**Completers (** ***N*** **=152)**							
QALY (EQ-5D)	324.9	−894.8	1,544.7	−0.00	−0.02	0.01	**TAU dominant**
EQ VAS	320.7	−894.3	1,535.6	2.67	0.56	4.79	**120**
**ITT (** ***N*** **=168)**							
QALY (EQ-5D)	145.9	−1,150.7	1,442.5	0.00	−0.01	0.02	**79,071**
EQ VAS	143.9	−1,150.8	1,438.6	2.72	0.42	5.02	**53**
**PPA (** ***N*** **=154)**							
QALY (EQ-5D)	181.4	−1,127.9	1,490.7	0.00	−0.01	0.02	**137,161**
EQ VAS	180.3	−1,127.3	1,487.9	2.65	0.36	4.94	**68**

The analysis of completers suggests that CBT is more cost-effective than TAU and RPT, as its incremental cost is significantly less than zero in both cases (approximately −2,100€ for CBT versus TAU and −2,400€ for CBT versus RPT), with the incremental effect on QALYs not being significant; however, they were very close to a 95% level in both cases. These findings are robust to different specifications of the sample, whether using the ITT-based or the PPA-based approach.

**Figure 2:**
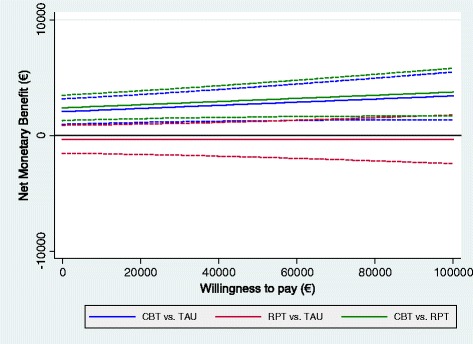
**Net-benefit curves, societal perspective; effectiveness measured on the EQ-5D based QALYs.** Dashed lines represent 95% confidence intervals.

Looking at the Net-Benefit (NB) curves plotted in Figure 2, we can see that the Net Monetary Benefit (NMB) and its 95% CIs for the CBT intervention are greater than zero at all hypothetical levels of willingness-to-pay (WTP) included.

**Figure 3:**
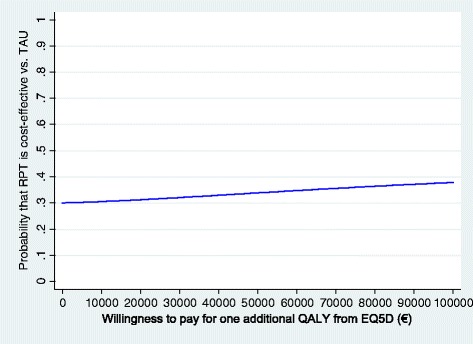
**Cost-effectiveness acceptability curve: RPT versus TAU; societal perspective; effectiveness measured on the EQ-5D-based QALYs.**

When looking at the comparison between RPT and TAU, findings from the completers’ analysis did not support the cost-effectiveness of the FDA-recommended intervention, as the average incremental effect for QALY was negative. The NMB for the RPT versus TAU comparison was negative at all WTPs, although the upper confidence interval was found to be consistently greater than zero. Looking at the related CEACs (Figure 3), we can see that at a WTP of 40,000€, RPT has a probability of only approximately 30% to be more cost-effective than TAU.

When using the EQ VAS as an outcome, the incremental cost remained significant, with an average value of −2,100€ for CBT versus TAU and approximately −2,400€ for CBT versus RPT. In this case, the incremental effect on EQ VAS was also found to be significant, with an average of approximately 3.5 points on the VAS (CBT versus RPT). The results found for this outcome reinforce the findings for QALYs. In the case of RPT versus TAU, we can see that the incremental cost is not significantly different from zero, but the incremental effect is greater than zero for all the three analyses (Completers, ITT, and PPA), which means that the medication treatment is able to improve HRQoL significantly with respect to usual care and is also likely to be cost-effective. The ICER was estimated at 120 for the completers’ analysis, estimated at 53 for the ITT analysis, and found to be 68 for the PPA case.

**Figure 4:**
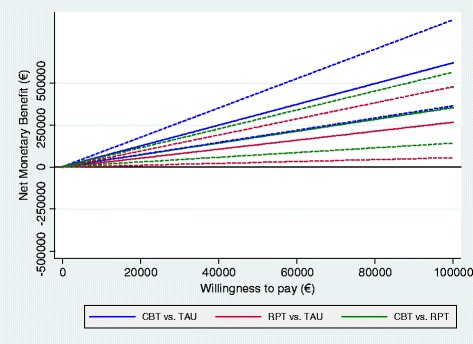
**Net benefit curves, societal perspective; effectiveness measured on the EQ VAS.** Dashed lines represent 95% confidence intervals.

**Figure 5:**
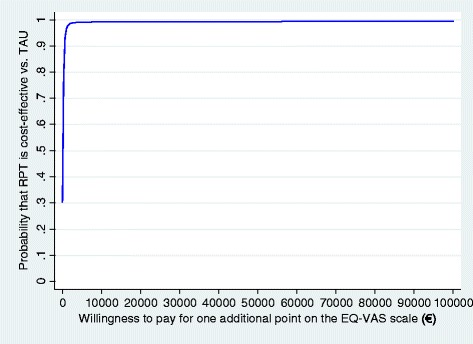
**Cost-effectiveness acceptability curve: RPT versus TAU; societal perspective; effectiveness measured on the EQ VAS scale.**

NB curves present a NMB consistently greater than zero; therefore, the analysis suggests that CBT is cost-effective against RPT and TAU at all the hypothesized WTP levels (see Figure 4). In Figure 5, we plotted the CEAC for RPT versus TAU, which suggests that the intervention is also likely to be cost-effective because of very small levels of WTP.

#### Healthcare perspective

The cost-effectiveness results from the healthcare perspective displayed in Table 6 were obtained by considering only the “direct healthcare costs”, including all the health and social care service uses, medications and intervention costs and excluding indirect costs. The findings are consistent with what has been found in the societal perspective. In particular, CBT has been found to be cost-effective with respect to TAU and with respect to RPT in the completers’ analysis and in the sensitivity analyses, when considering EQ-5D based QALYs and EQ VAS. By using EQ-5D-based QALYs as the outcome, we found that TAU dominates RPT when using the completers’ analysis perspective, whereas in the ITT and PPA cases, ICERs were set to approximately 100,000€, which is well above established cost-effectiveness thresholds.

**Table 6:** **Incremental cost-effectiveness ratios, healthcare perspective, 0 to 6 months**

	**Incremental cost**			**Incremental effect**			**ICER**
	**Mean; (95% bootstrap CI)**			**Mean; (95% bootstrap CI)**			
**CBT versus TAU**							
**Completers (** ***N*** **=152)**							
QALY (EQ-5D)	−1,577.6	−2,601.8	−553.4	0.01	−0.00	0.03	**CBT dominant**
EQ VAS	−1,583.8	−2,606.9	−560.7	6.19	3.63	8.75	**CBT dominant**
**ITT (** ***N*** **=168)**							
QALY (EQ-5D)	−1,632.6	−2,744.6	−520.7	0.02	−0.00	0.03	**CBT dominant**
EQ VAS	−1,635.0	−2,747.6	−522.3	6.33	3.59	9.07	**CBT dominant**
**PPA (** ***N*** **=154)**							
QALY (EQ-5D)	−1,773.5	−2,924.9	−622.1	0.02	−0.00	0.03	**CBT dominant**
EQ VAS	−1,775.1	−2,927.6	−622.7	6.58	3.83	9.33	**CBT dominant**
**CBT versus RPT**							
**Completers (** ***N*** **=152)**							
QALY (EQ-5D)	−1,866.8	−2,917.6	−816.0	0.01	−0.00	0.03	**CBT dominant**
EQ VAS	−1,870.8	−2,921.6	−819.9	3.52	1.13	5.91	**CBT dominant**
**ITT (** ***N*** **=168)**							
QALY (EQ-5D)	−1,804.0	−2,786.7	−821.3	0.01	−0.00	0.03	**CBT dominant**
EQ VAS	−1,805.4	−2,788.6	−822.2	3.61	1.08	6.15	**CBT dominant**
**PPA (** ***N*** **=154)**							
QALY (EQ-5D)	−1,972.8	−3,008.2	−937.5	0.01	−0.00	0.03	**CBT dominant**
EQ VAS	−1,973.7	−3,011.3	−936.1	3.93	1.35	6.52	**CBT dominant**
**RPT versus TAU**							
**Completers (** ***N*** **=152)**							
QALY (EQ-5D)	289.2	−858.1	1,436.5	−0.00	−0.02	0.01	**TAU dominant**
EQ VAS	287.0	−857.3	1,431.2	2.67	0.55	4.79	**107**
**ITT (** ***N*** **=168)**							
QALY (EQ-5D)	171.4	−1,037.3	1,380.0	0.00	−0.01	0.02	**98,434**
EQ VAS	170.4	−1,037.1	1,377.9	2.72	0.42	5.02	**63**
**PPA (** ***N*** **=154)**							
QALY (EQ-5D)	199.3	−1,023.0	1,421.6	0.00	−0.01	0.02	**107,697**
EQ VAS	198.6	−1,022.6	1,419.7	2.65	0.35	4.94	**75**

When using EQ VAS as the outcome, we found that the incremental cost is not significantly different from zero, but with the incremental effect being greater than zero, there are hints that the RPT may be cost-effective with respect to usual care. These findings remain consistent when observing at the sensitivity analyses that were implemented, with ICERs ranging from 63 to 107.

## Discussion

This article extends our previous findings [23], demonstrating for the first time that a group-based form of CBT is more cost-effective for the treatment of FM than usual care and FDA-recommended drugs. The results of the economic evaluation can be summarized as follows: As expected, FM syndrome is associated with high healthcare and societal costs, which is in accordance with several previous studies conducted across different FM samples from different countries [5,15]. Focusing on Spain, the baseline evaluation indicated that our FM patients from Aragon produced higher healthcare costs (1,940€) and sick-leave costs (810€), on average, than those reported by Sicras-Mainar *et al.* [46] in Catalonia (healthcare costs = 1,677€ and sick-leave costs = 815.8€); of note is the difference in timeframes (6-month period versus 12-month period). The disparities in costs between both Spanish studies might be due to the inclusion of a different range of health care services or to structural differences in the health care systems of these Spanish regions but are not likely to be caused by significant differences in functional status, given that our participants, as reported in a previous article [23], were apparently less impaired (Alda *et al*. [23]: Fibromyalgia Impact Questionnaire score = 65.6 versus Sicras-Mainar *et al.* [46]: Fibromyalgia Impact Questionnaire score = 71.7).

The QALY differences between the CBT group and the RPT and TAU groups observed in our sample (0.02 and 0.01, respectively) were smaller than those previously reported in economic evaluations of nonpharmacologic treatments (for example, Luciano *et al*. [21]: QALY difference in usual care *plus* psycho-education versus usual care = 0.12; Gusi and Tomás-Carús [21]: QALY difference in usual care *plus* exercise versus usual care = 0.13). Any clinical interpretation (for example, greater effectiveness of psycho-education or exercise compared with CBT) of the differences might be considered speculative.

At the 6-month follow-up, CBT was dominant from both healthcare and societal perspectives compared with RPT and TAU. The examination of the NB curves (with the CI curves lying above the x-axis) permits us to conclude that the CBT intervention might be considered cost-effective, not only in our country, but also for any European or North-American policymaker taking current country-specific investment ceilings into account (for example, Netherlands = €30,000/QALY; UK = £30,000/QALY; USA = $60,000/QALY).

Our study is thought to be the first that supports CBT as a cost-effective treatment option for FM patients, as determined by a head-to-head comparison against usual care and FDA-recommended drugs. Findings were consistent by using EQ-5D based QALYs and EQ VAS as outcomes and from societal and healthcare perspectives. Moreover, the robustness of the results was confirmed by exploring the variation in methodologic approaches (ITT and PPA sensitivity analyses). In the current context of the economic crisis in Spain, in which the healthcare system has scant resources to attend to patients with chronic pain, we believe that the findings of this full economic evaluation might be interesting for policymakers. Bearing in mind the present data and the results recently published by Luciano *et al*. [22] regarding the long-term cost-utility of a 2-month psycho-educational program compared with usual care, it might be advisable to establish an IAPT program (*Improving Access to Psychological Therapies program*) in Spain for patients with chronic pain conditions, like the program that already exists in the UK for patients with common mental health problems [47].

Notwithstanding this, although our CBT program has proven to be cost-effective, policymakers will need further information concerning the investment needed to train new CBT therapists. What would be the cost of implementing group CBT in everyday medical practice? Currently, not all first-line clinicians (GPs and nurses) or specialists (clinical psychologists, psychiatrists, rheumatologists, and so on) in Spain have experience or training in group-based forms of CBT. Moreover, it is well known that certain factors, such as the therapist’s level of expertise, may account for the psychological improvements observed after psychological interventions. Finding clinicians who are well-trained in CBT and who have experience in group psychotherapy is not an easy task. In our opinion, future studies should model the long-term impact of CBT training on cost-effectiveness.

When comparing RPT and TAU, we found evidence that the former option is cost-effective, but only when using EQ VAS as an outcome. When looking at the ITT analysis from the societal perspective, we found an ICER of approximately 80,000€ that could be interesting for policymakers, although it is far from the commonly adopted thresholds. At this point, it is important to indicate that the GPs of patients included in the TAU group had received the Guide for the Treatment of FM in Primary Care elaborated by the Aragonese Health Service, which recommends pregabalin and duloxetine as the first pharmacologic treatment option. Indeed, of the 49 patients in the TAU group that were followed up for economic evaluation at 6 months, 43 (88%) had taken anticonvulsants (25 pregabalin) and 40 (82%), antidepressants (19 duloxetine), throughout the study. This aspect could explain the nonsignificant differences in costs and in the effectiveness between the RPT and TAU groups.

From our point of view, the modest and uncertain superiority of FDA-recommended drugs over usual care is not surprising. Although a network meta-analysis [48] indicated the significant efficacy of pregabalin and SNRIs (duloxetine and milnacipran) against placebo for the treatment of FM, recent observational studies with large samples have seriously questioned their efficacy in routine practice. One such study is REFLECTIONS (Real World Examination of Fibromyalgia: Longitudinal Evaluation of Costs and Treatments) [49], a prospective 12-month observational study of adult patients with fibromyalgia (*N* = 1,700) who started a new pharmacologic treatment among four medication cohorts (pregabalin, duloxetine, milnacipran, and TCAs). Patients observed reported statistically significant but nonclinically relevant improvements from baseline to each follow-up visit. Wolfe *et al*. [50] examined the changes in pharmacologic therapies that have occurred over the last decade in a wide sample of adult FM patients (*N* = 3,123). Regarding the effect of treatment with FDA-recommended drugs on FM outcomes, the authors observed that the changing patterns of FM pharmacotherapy (from nonsteroidal antiinflammatory drugs and opioids to FDA drugs) did not change the outcomes of pain, fatigue, or functional status. In other words, switching to FDA-recommended drugs (which are more expensive) did not result in clinically relevant benefits.

Some strengths of the present work should be highlighted: the use of an RCT design with a long follow-up period that permitted the capture of changes in costs and outcomes; the economic evaluation that followed the recent CHEERS statement [28] for reporting economic evaluations of health interventions; a comprehensive interpretation and reporting of data that provides not only statistical significance tests but also addresses the associated uncertainty; additionally, as we mentioned earlier, Bernardy *et al*. [14] assigned a high score to our trial (7 points) by using Yates rating scale [29], which is indicative of the considerable treatment quality; finally, the usual care condition (TAU) reflects current clinical practice in the context of public health care in Aragon (Spain), providing a meaningful comparison to determine the added value of the CBT group intervention and its potential inclusion in future clinical guidelines in this Spanish region. Nonetheless, our findings require replication in other Spanish regions or European countries before firm conclusions can be established regarding the superiority of group CBT compared with FDA-recommended treatment and usual care.

We acknowledge the following study limitations and shortcomings; first, it is under debate whether the use of EQ-5D is appropriate in the economic evaluation of psychological treatments because it represents a generic measure of health status, with one of its dimensions capturing a psychological construct (anxiety/depression). However, according to Onrust *et al*. [51], if a participant reports experiencing “extreme problems” on anxiety/depression without any other health problems, the health status would be evaluated as “poor”. Therefore, only by reducing the mental health problems from “extreme” to a better outcome, psychological treatments would be able to improve patients’ EQ-5D utility score. Furthermore, although medical procedures different from psychological treatments may be able to give a substantial improvement in QALYs, their associated costs are usually much higher as well. Psychological treatments do not need to produce large changes in QALYs to be cost-effective. Overall, although we agree with the remarks from Onrust *et al*. [51], we add that our case seems more favorable, as CBT intervention was associated both with improvements in QALYs (although nonsignificant) and with statistically significant reductions in direct and indirect costs.

Second, among the other limitations, we can mention the lack of an active nonpharmacologic control group. We have not compared the cost-effectiveness of CBT with other second- or third-generation psychological treatments and nonpharmacologic options that are available in a group format and that have proven to be effective for improving the functional status and quality of life of FM patients, such as Acceptance and Commitment Therapy [52], Mindfulness-Based Stress Reduction [53], or aerobic exercise [54]. In our opinion, future RCTs should compare the cost-effectiveness of CBT with other active nonpharmacologic control conditions that are equivalent to the CBT intervention in therapy time and therapist allegiance. Indeed, we think that the lack of control over therapist allegiance is one of the main flaws of the current RCT. According to Nuesch *et al.* [11], a combination of pregabalin or SNRIs, as pharmacologic interventions, and multicomponent therapy, aerobic exercise, and CBT, as nonpharmacologic interventions, seem most promising for the management of FM symptoms. From our point of view, it might have been very interesting to examine the effectiveness and cost-effectiveness of a combined therapy (for example, CBT as add-on to FDA-recommended drugs) compared with either of our treatments alone [55].

Third, direct costs were most likely underestimated in our study because of the absence of direct non-healthcare costs (for example, patients’ travel costs to the healthcare center or temporary hired caregivers). Similarly, regarding indirect costs, we did not assess productivity losses associated with reduced efficiency at work (absenteeism) and with unpaid work (for example, household work) because methodologic difficulties are involved in measuring these losses, and the size of the difference between study groups was expected to be minimal. Benefit payments for disability or unemployment (transfer costs), included in some cost-of-illness studies, were excluded from our economic evaluation, given that they represent an accounting cost to the government, but not to the society as a whole.

Fourth, between-group differences in treatment expectations and treatment credibility were not assessed at baseline, which supposes a significant threat to our study's internal validity. Smeets *et al*. [56] demonstrated that chronic lower-back-pain patients' initial expectations and credibility about the benefits of pain treatment had an influence on the final treatment outcome. In our opinion, future studies should address the predictive capacity of treatment expectations and treatment credibility on the cost-effectiveness results.

Fifth, another limitation arises from the fact that we used economic tariffs instead of unit costs because these are not published unit costs in Spain. Economic tariffs may differ slightly from real unit costs.

Finally, some previous studies have involved family members in the psychological treatment of children/adolescents with chronic conditions, yielding promising results [57]. In our study, spouses/significant others of the CBT participants were not included in the treatment (only a minimal participation of spouses in session 7). Future studies might analyze whether involving spouses or significant others in group CBT for FM syndrome is more effective than standard group CBT.

## Conclusions

In summary, the results of the present work support that group CBT as a stand-alone intervention is cost-effective compared with FDA-recommended drugs and usual care. Therefore, a wider implementation of CBT programs in group format for FM patients within the public provision of healthcare in Spain is recommended.

## Endnotes

^a^Alda *et al*. [23]: CBT significantly decreased global pain catastrophizing (primary end point) at the 6-month follow-up examination with effect sizes of the Cohen *d* = 0.73 and 1.01 compared with RPT and TAU, respectively. CBT was also more effective than RPT and TAU at increasing pain acceptance, functional status, and HRQoL. No differences were noted among the three treatments with regard to pain and depression.

^b^Although the cost-utility analysis was not described in the ISRCTN register (http://www.controlled-trials.com/ISRCTN10804772/), it was planned *a priori*. The analysis strategy of the economic evaluation was briefly explained in the study protocol published by García-Campayo and collaborators [25].

## Additional file

Additional file 1:
**Consolidated Health Economic Evaluation Reporting Standards (CHEERS) statement.** Completed CHEERS checklist.

## Abbreviations

CBT: Cognitive behavioral therapy
CEAC: cost-effectiveness acceptability curve
CONSORT: consolidated standards of reporting trials guidelines
CSRI: client service receipt inventory
EQ-5D: EuroQol 5-D questionnaire
FDA: US Food and Drug Administration
FM: fibromyalgia
HRQOL: Health-related quality of life
ICER: incremental cost-effectiveness ratio
ITT: intention-to-treat analysis
PPA: per protocol analysis
QALY: quality-adjusted life years
RPT: FDA-recommended pharmacologic treatment
SNRI: serotonin–norepinephrine reuptake inhibitor
TAU: treatment as usual
